# Privacy-preserving logistic regression training

**DOI:** 10.1186/s12920-018-0398-y

**Published:** 2018-10-11

**Authors:** Charlotte Bonte, Frederik Vercauteren

**Affiliations:** imec-Cosic, Dept. Electrical Engineering, KU Leuven, Kasteelpark Arenberg 10, Leuven, Belgium

**Keywords:** Homomorphic encryption, Logistic regression, Privacy, Fixed Hessian

## Abstract

**Background:**

Logistic regression is a popular technique used in machine learning to construct classification models. Since the construction of such models is based on computing with large datasets, it is an appealing idea to outsource this computation to a cloud service. The privacy-sensitive nature of the input data requires appropriate privacy preserving measures before outsourcing it. Homomorphic encryption enables one to compute on encrypted data directly, without decryption and can be used to mitigate the privacy concerns raised by using a cloud service.

**Methods:**

In this paper, we propose an algorithm (and its implementation) to train a logistic regression model on a homomorphically encrypted dataset. The core of our algorithm consists of a new iterative method that can be seen as a simplified form of the fixed Hessian method, but with a much lower multiplicative complexity.

**Results:**

We test the new method on two interesting real life applications: the first application is in medicine and constructs a model to predict the probability for a patient to have cancer, given genomic data as input; the second application is in finance and the model predicts the probability of a credit card transaction to be fraudulent. The method produces accurate results for both applications, comparable to running standard algorithms on plaintext data.

**Conclusions:**

This article introduces a new simple iterative algorithm to train a logistic regression model that is tailored to be applied on a homomorphically encrypted dataset. This algorithm can be used as a privacy-preserving technique to build a binary classification model and can be applied in a wide range of problems that can be modelled with logistic regression. Our implementation results show that our method can handle the large datasets used in logistic regression training.

## Background

### Introduction

Logistic regression is a popular technique used in machine learning to solve binary classification problems. It starts with a training phase during which one computes a model for prediction based on previously gathered values for predictor variables (called covariates) and corresponding outcomes. The training phase is followed by a testing phase that assesses the accuracy of the model. To this end, the dataset is split into data for training and data for validation. This validation is done by evaluating the model in the given covariates and comparing the output with the known outcome. When the classification of the model equals the outcome for most of the test data, the model is considered to be valuable and it can be used to predict the probability of an outcome by simply evaluating the model for new measurements of the covariates.

Logistic regression is popular because it provides a simple and powerful method to solve a wide range of problems. In medicine, logistic regression is used to predict the risk of developing a certain disease based on observed characteristics of the patient. In politics, it is used to predict the voting behaviour of a person based on personal data such as age, income, sex, state of residence, previous votes. In finance, logistic regression is used to predict the likelihood of a homeowner defaulting on a mortgage or a credit card transaction being fraudulent.

As all machine learning tools, logistic regression needs sufficient training data to construct a useful model. As the above examples show, the values for the covariates and the corresponding outcomes are typically highly sensitive, which implies that the owners of this data (either people or companies) are reluctant to have their data included in the training set. In this paper, we solve this problem by describing a method for privacy preserving logistic regression *training* using somewhat homomorphic encryption. Homomorphic encryption enables computations on encrypted data without needing to decrypt the data first. As such, our method can be used to send encrypted data to a central server, which will then perform logistic regression training on this encrypted input data. This also allows to combine data from different data owners since the server will learn nothing about the underlying data.

### Related work

Private logistic regression with the aid of homomorphic encryption has already been considered in [1, 2], but in a rather limited form: both papers assume that the logistic model has already been trained and is publicly available. This publicly known model is then evaluated on homomorphically encrypted data in order to perform classification of this data without compromising the privacy of the patients. Our work complements these works by executing the training phase for the logistic regression model in a privacy-preserving manner. This is a much more challenging problem than the classification of new data, since this requires the application of an iterative method and a solution for the nonlinearity in the minimization function.

Aono et al. [3] also explored secure logistic regression via homomorphic encryption. However, they shift the computations that are challenging to perform homomorphically to trusted data sources and a trusted client. Consequently, in their solution the data sources need to compute some intermediate values, which they subsequently encrypt and send to the computation server. This allows them to only use an additively homomorphic encryption scheme to perform the second, easier, part of the training process. Finally, they require a trusted client to perform a decryption of the computed coefficients and use these coefficients to construct the cost function for which the trusted client needs to determine the minimum in plaintext space. Their technique is based on a polynomial approximation of the logarithmic function in the cost function and the trusted client applies the gradient descent algorithm as iterative method to perform the minimization of the cost function resulting from the homomorphic computations. Our method does not require the data owners to perform any computations (bar the encryption of their data) and determines the model parameters by executing the minimization directly on encrypted data. Again this is a much more challenging problem.

In [4] Xie et al. construct PrivLogit which performs logistic regression in a privacy-preserving but distributed manner. As before, they require the data owners to perform computations on their data before encryption to compute parts of a matrix used in the logistic regression. Our solution starts from the encrypted raw dataset, not from values that were precomputed by the centers that collect the data. In our solution all computations that are needed to create the model parameters, are performed homomorphically.

Independently and in parallel with our research, Kim et al. [5] investigated the same problem of performing the training phase of logistic regression in the encrypted domain. Their method uses a different approach than ours: firstly, they use a different minimization method (gradient descent) compared to ours (a simplification of the fixed Hessian method), a different approximation of the sigmoid function and a different homomorphic encryption scheme. Their solution is based on a small adaptation of the input values, which reduces the number of homomorphic multiplications needed in the computation of the model. We assumed the dataset would be already encrypted and therefore adaptations to the input would be impossible. Furthermore, they tested their method on datasets that contain a smaller number of covariates than the datasets used in this article.

### Contributions

Our contributions in this paper are as follows: firstly, we develop a method for privacy preserving logistic training using homomorphic encryption that consists of a low depth version of the fixed Hessian method. We show that consecutive simplifications result in a practical algorithm, called the simplified fixed Hessian (SFH) method, that at the same time is still accurate enough to be useful. We implemented this algorithm and tested its performance and accuracy on two real life use cases: a medical application predicting the probability of having cancer given genomic data and a financial application predicting the probability that a transaction is fraudulent. Our test results show that in both use cases the model computed is almost as accurate as the model computed by standard logistic regression tools such as the ones present in Matlab.

## Technical Background

### Logistic regression

Logistic regression can be used to predict the probability that a dependent variable belongs to a class, e.g. healthy or sick, given a set of covariates, e.g. some genomic data. In this article, we will consider binary logistic regression, where the dependent variable can belong to only two possible classes, which are labelled {±1}. Binary logistic regression is often used for binary classification by setting a threshold for a given class up front and comparing the output of the regression with this threshold. The logistic regression model is given by:

1 $$ \text{Pr}(y=\pm1|\mathbf{x},\boldsymbol{\beta})=\sigma\left(y\boldsymbol{\beta}^{T}\mathbf{x}\right)=\frac{1}{1+e^{\left(-y\boldsymbol{\beta}^{T}\mathbf{x}\right)}} \,,  $$

where the vector ***β***=(*β*_0_,…,*β*_*d*_) are the model parameters, *y* the class label (in our case {±1}) and the vector $\mathbf {x} = (1, x_{1}, \ldots, x_{d}) \in \mathbb {R}^{d+1}$ the covariates.

Because logistic regression predicts probabilities rather than classes, we can generate the model using the log likelihood function. The training of the model starts with a training dataset (**X**,**y**)=[(*x*_1_,*y*_1_),…,(*x*_*N*_,*y*_*N*_)], consisting of *N* training vectors $\mathbf {x_{i}}=(1,x_{i,1},\ldots,x_{i,d})~\in ~\mathbb {R}^{d+1}$ and corresponding observed class *y*_*i*_ ∈ {−1,1}. The goal is to find the parameter vector ***β*** that maximizes the log likelihood function:

2 $$ l(\boldsymbol{\beta})=-\sum_{i=1}^{n}{\log\left(1+e^{\left(-y_{i}\boldsymbol{\beta}^{T}\mathbf{x_{i}}\right)}\right)}\,.  $$

When the parameters ***β*** are determined, they can be used to classify new data vectors $\mathbf {x^{\text {new}}}=\left (1,x^{\text {new}}_{1},\ldots, x^{\text {new}}\right)~\in ~\mathbb {R}^{d+1}$ by setting

$$y^{\text{new}}=\left\{\begin{array}{ll} 1 &\text{if}\;p\left(y=1|\mathbf{x^{\text{new}}},\boldsymbol{\beta}\right) \geq \tau\\ -1 &\quad\text{if}\;p\left(y=1|\mathbf{x^{\text{new}}},\boldsymbol{\beta}\right) < \tau\\ \end{array}\right. $$

in which 0<*τ*<1 is a variable threshold which typically equals $\frac {1}{2}$.

### Datasets

As mentioned before, we will test our method in the context of two real life use cases, one in genomics and the other in finance.

The genomic dataset was provided by the iDASH competition of 2017 and consists of 1581 records (each corresponding to a patient) consisting of 103 covariates and a class variable indicating whether or not the patient has cancer. The challenge was to devise a logistic regression model to predict the disease given a training data set of at least 200 records and 5 covariates. However, for scalability reasons the solution needed to be able to scale up to 1000 records with 100 covariates. This genomic dataset consists entirely of binary data.

The financial data was provided by an undisclosed bank that provided anonymized data with the goal of predicting fraudulent transactions. Relevant data fields that were selected are: type of transaction, effective amount of the transaction, currency, origin and destination, fees and interests, etc. This data has been subject to preprocessing by firstly representing the non-numerical values with labels and secondly computing the minimum and maximum for each of the covariates and using these to normalise the data by computing $\frac {x-x_{\text {min}}}{x_{\text {max}}-x_{\text {min}}}$. The resulting financial dataset consists of 20,000 records with 32 covariates, containing floating point values between 0 and 1.

### The FV scheme

Our solution is based on the somewhat homomorphic encryption scheme of Fan and Vercauteren [6], which can be used to compute a limited number of additions and multiplications on encrypted data. The security of this encryption scheme is based on the hardness of the ring learning with error problem (RLWE) introduced by Lyubashevsky et al. in [7]. The core objects in the FV scheme are elements of the polynomial ring $R=\mathbb {Z}[X]\slash (f(X))$, where typically one chooses *f*(*X*)=*X*^*D*^+1 for *D*=2^*n*^ (in our case *D*=4096). For an integer modulus $M \in \mathbb {Z}$ we denote with *R*_*M*_ the quotient ring *R*/(*MR*).

The plaintext space of the FV scheme is the ring *R*_*t*_ for *t*>1 a small integer modulus and the ciphertext space is *R*_*q*_×*R*_*q*_ for an integer modulus *q*≫*t*. For *a*∈*R*_*q*_, we denote by [*a*]_*q*_ the element in *R* obtained by applying [·]_*q*_ to all its coefficients *a*_*i*_, with [*a*_*i*_]_*q*_=*a*_*i*_ mod *q* given by a representative in $\left (\frac {-q}{2},\frac {q}{2}\right ]$. The FV scheme uses two probability distributions on *R*_*q*_: one is denoted by *χ*_key_ and is used to sample the secret key of the scheme, the other is denoted *χ*_err_ and will be used to sample error polynomials during encryption. The exact security level of the FV scheme is based on these probability distributions, the degree *D* and the ciphertext modulus *q* and can be determined using an online tool developed by Albrecht et al. [8].

Given parameters *D*, *q*, *t* and the distributions *χ*_key_ and *χ*_err_, the core operations are then as follows:

- KeyGen: the private key consists of an element *s*←*χ*_key_ and the public key **pk**=(*b*,*a*) is computed as *a*←*R*_*q*_ uniformly at random and *b*=[−(*as*+*e*)]_*q*_ with *e*←*χ*_err_.
- Encrypt(**pk**, *m*): given *m*∈*R*_*t*_, sample error polynomials *e*_1_,*e*_2_∈*χ*_*err*_ and *u*∈*χ*_*key*_ and compute *c*_0_=*Δm*+*bu*+*e*_1_ and *c*_1_=*au*+*e*_2_ with *Δ*=⌊*q*/*t*⌋, the largest integer smaller than $\frac {q}{t}$. The ciphertext is then **c**=(*c*_0_,*c*_1_).
- Decrypt(sk, **c**): compute $\tilde {m}=[c_{0}+c_{1} s]_{q}$, divide the coefficients of $\tilde {m}$ by *Δ* and round and reduce the result into *R*_*t*_.

Computing the sum of two ciphertexts simply amounts to adding the corresponding polynomials in the ciphertexts. Multiplication, however, requires a bit more work and we refer to [6] for the precise details.

The relation between a ciphertext and the underlying plaintext can be described as [*c*_0_+*c*_1_*s*]_*q*_=*Δm*+*e*, where *e* is the noise component present in the ciphertext. This also shows that if the noise *e* grows too large, decryption will no longer result in the original message, and the scheme will no longer be correct. Since the noise present in the resulting ciphertext will grow with each operation we perform homomorphically, it is important to choose parameters that guarantee correctness of the scheme. Knowing the computations that need to be performed up front enables us to estimate the size of the noise in the resulting ciphertext, which permits the selection of suitable parameters.

### w-NIBNAF

In order to use the FV scheme, we need to transform the input data into polynomials of the plaintext space *R*_*t*_. To achieve this, our solution makes use of the *w*-NIBNAF encoding, because this encoding improves the performance of the homomorphic scheme. The *w*-NIBNAF encoding is introduced in [9] and expands a given number *θ* with respect to a non-integral base 1<*b*_*w*_<2. By replacing the base *b*_*w*_ by the variable *X*, the method encodes any real number *θ* as a Laurent polynomial:

3 $$ \begin{aligned} \theta =& a_{r}X^{r}+a_{r-1}X^{r-1}+\dotsb+a_{1}X+a_{0}-a_{-1}X^{d-1}\\ &-a_{-2}X^{d-2}-\dotsb-a_{-s}X^{d-s}. \end{aligned}  $$

A final step then maps this Laurent polynomial into the plaintext space *R*_*t*_ and we refer the reader to [9] for the precise details.

The *w*-NIBNAF encoding is constructed such that the encoding of a number will satisfy two conditions: the encoding has coefficients in the set {−1,0,1} and each set of *w* consecutive coefficients will have no more than one non-zero coefficient. Both conditions ensure that the encoded numbers are represented by very sparse polynomials with coefficients in the set {−1,0,1}, which can be used to bound the size of the coefficients of the result of computations on these representations. In particular, this encoding results in a smaller plaintext modulus *t*, which improves the performance of the homomorphic encryption scheme. Since larger values for *w* increase the sparseness of the encodings and hence reduce the size of *t* even more, one would like to select the value for *w* to be as large as possible. However, similar to encryption one has to consider a correctness requirement for the encoding. More specifically, decoding of the final polynomial should result in the correct answer, hence the base *b*_*w*_ and consequently also the value of *w* should be chosen with care.

## Methods

### Privacy preserving training of the model

#### Newton-Raphson method

To estimate the parameters of our logistic regression model, we need to compute the parameter vector ***β*** that maximizes Eq. (2). Typically, one would differentiate the log likelihood equation with respect to the parameters, set the derivatives equal to zero and solve these equations to find the maximum. The gradient of the log likelihood function *l*(***β***), i.e. the vector of its partial derivatives [*∂l*/*∂β*_0_, *∂l*/*∂β*_1_, …, *∂l*/*∂β*_*d*_] is given by:

$$\nabla_{\boldsymbol{\beta}}l(\boldsymbol{\beta})=\sum_{i}{\left(1-\sigma\left(y_{i}\boldsymbol{\beta}^{T}\mathbf{x}_{i}\right)\right)y_{i}\mathbf{x}_{i}}\,. $$

In order to estimate the parameters ***β***, this equation will be solved numerically by applying the Newton-Raphson method, which is a method to numerically determine the zeros of a function. The iterative formula of the Newton-Raphson method to calculate the root of a univariate function *f*(*x*) is given by:

4 $$ x_{k+1} = x_{k} -\frac{f(x_{k})}{f'(x_{k})}\,,  $$

with *f*^′^(*x*) the derivative of *f*(*x*). Since we now compute with a multivariate objective function *l*(***β***), the (*k*+1)^th^ iteration for the parameter vector ***β*** is given by:

5 $$ \boldsymbol{\beta}_{k+1}=\boldsymbol{\beta}_{k}-H^{-1}\left(\boldsymbol{\beta}_{k}\right)\nabla_{\boldsymbol{\beta}}l\left(\boldsymbol{\beta}_{k}\right) \,,  $$

with ∇_***β***_*l*(***β***) as defined above and $H(\boldsymbol {\beta })=\nabla ^{2}_{\boldsymbol {\beta }}l(\boldsymbol {\beta })$ the Hessian of *l*(***β***), being the matrix of its second partial derivatives *H*_*i*,*j*_=*∂*^2^*l*/*∂β*_*i*_*∂β*_*j*_, given by:

$$H(\boldsymbol{\beta})=-\sum_{i}{\left(1-\sigma\left(y_{i}\boldsymbol{\beta}^{T}\mathbf{x}_{i}\right)\right)\sigma\left(y_{i}\boldsymbol{\beta}^{T}\mathbf{x}_{i}\right)\left(y_{i}\mathbf{x}_{i}\right)^{2}}\,. $$

#### Homomorphic logistic regression

The downside of Newton’s method is that exact evaluation of the Hessian and its inverse are quite expensive in computational terms. In addition, the goal is to estimate the parameters of the logistic regression model in a privacy-preserving manner using homomorphic encryption, which will further increase the computational challenges. Therefore, we will adapt the method in order to make it possible to compute it efficiently in the encrypted domain.

The first step in the simplification process is to approximate the Hessian matrix with a fixed matrix instead of updating it every iteration. This technique is called the fixed Hessian Newton method. In [10], Böhning and Lindsay investigate the convergence of the Newton-Raphson method and show it converges if the Hessian *H*(***β***) is replaced by a fixed symmetric negative definite matrix *B* (independent of ***β***) such that *H*(***β***)≥*B* for all feasible parameter values ***β***, where “ ≥” denotes the Loewner ordering. The Loewner ordering is defined for symmetric matrices *A*, *B* and denoted as *A*≥*B* iff their difference *A*−*B* is non-negative definite. Given such *B*, the Newton-Raphson iteration simplifies to

$$\boldsymbol{\beta}_{k+1}=\boldsymbol{\beta}_{k}-B^{-1}\nabla_{\boldsymbol{\beta}}l(\boldsymbol{\beta}_{k})\,. $$

Furthermore, they suggest a lower bound specifically for the Hessian of the logistic regression problem, which is defined as $\bar {H} = -\frac {1}{4} X^{T}X$ and demonstrate that this is a good bound. This approximation does not depend on ***β***, consequently it is fixed throughout all iterations and it only needs to be computed once as desired. Since the Hessian is fixed, so is its inverse, which means it only needs to be computed once.

In the second step, we will need to simplify this approximation even more, since inverting a square matrix whose dimensions equal the number of covariates (and thus can be quite large), is nearly impossible in the encrypted domain. To this end, we replace the matrix $\bar {H}$ by a diagonal matrix for which the method still converges. The entries of the diagonal matrix are simply the sums of the rows of the matrix $\bar {H}$, so our new approximation $\tilde {H}$ of the Hessian becomes:

$$\tilde{H}=\left[ \begin{array}{cccc} {\sum\nolimits}_{i=0}^{d}{\bar{h}_{0,i}} & 0 & \ldots & 0\\ 0 & {\sum\nolimits}_{i=0}^{d}{\bar{h}_{1,i}} & \ldots & 0\\ \vdots & \vdots & \ddots & \vdots\\ 0 & 0 & \ldots & {\sum\nolimits}_{i=0}^{d}{\bar{h}_{d,i}}\\ \end{array} \right]\,. $$

To be able to use this approximation as lower bound for the above fixed Hessian method we need to assure ourselves it satisfies the condition $H(\boldsymbol {\beta })\geq \tilde {H}$. As mentioned before we already know from [10] that $H(\boldsymbol {\beta })\geq \frac {-1}{4}X^{T}X$, so it is sufficient to show that $\frac {-1}{4}X^{T}X\geq \tilde {H}$, which we now prove more generally.

##### **Lemma 1**

Let $A \!\in \! \mathbb {R}^{n \times n}$ be a symmetric matrix with all entries non-positive, and let *B* be the diagonal matrix with diagonal entries $B_{k,k} \,=\, \sum _{i = 1}^{n} A_{k,i}$ for *k* = 1,…,*n*, then *A* ≥ *B*.

##### *Proof*

By definition of the matrix *B*, we have that *C*=*A*−*B* has the following entries: for *i*≠*j* we have *C*_*i*,*j*_=*A*_*i*,*j*_ and $C_{i,i} = - \sum _{k = 1, k \neq i}^{n} A_{i,k}$. In particular, the diagonal elements of *C* are minus the sum of the off-diagonal elements on the *i*-th row. We can bound the eigenvalues *λ*_*i*_ of *C* by Gerschgorin’s circle theorem [11], which states that for every eigenvalue *λ* of *C*, there exists an index *i* such that

$$|\lambda - C_{i,i}| \leq \sum_{j\neq i}{|C_{ij}|} \qquad i\in\{1,2,\ldots,n\} \,. $$

Note that by construction of *C* we have that $C_{i,i} = \sum _{j\neq i}{|C_{ij}|}$, and so every eigenvalue *λ* satisfies |*λ*−*C*_*i*,*i*_|<*C*_*i*,*i*_ for some *i*. In particular, since *C*_*i*,*i*_≥0, we conclude that *λ*≥0 for all eigenvalues *λ* and thus that *A*≥*B*. □

Our approximation $\tilde {H}$ for the Hessian also simplifies the computation of the inverse of the matrix, since we simply need to invert each diagonal element separately. The inverse will be again computed using the Newton-Raphson method: assume we want to invert the number *a*, then the function *f*(*x*) will be equal to $\frac {1}{x}-a$ and the iteration is given by *x*_*k*+1_=*x*_*k*_(2−*ax*_*k*_). For the Newton-Raphson method to converge, it is important to determine a good start value. Given the value range of the input data and taking into account the dimensions of the training data, we estimate a range of the size of the number we want to invert. This results in an estimation of the order of magnitude of the solution that is expected to be found by the Newton-Raphson algorithm. By choosing the initial value of our Newton-Raphson iteration close to the constructed estimation of the inverse, we can already find an acceptable approximation of the inverse by performing only one iteration of the method.

In the third and final step, we simplify the non-linearity coming from the sigmoid function. Here, we simply use the Taylor series: extensive experiments with plaintext data showed that approximating *σ*(*y*_*i*_***β***^*T*^**x**_*i*_) by $\frac {1}{2}+\frac {y_{i}\boldsymbol {\beta }^{T}\mathbf {x}_{i}}{4}$ is enough to obtain good results.


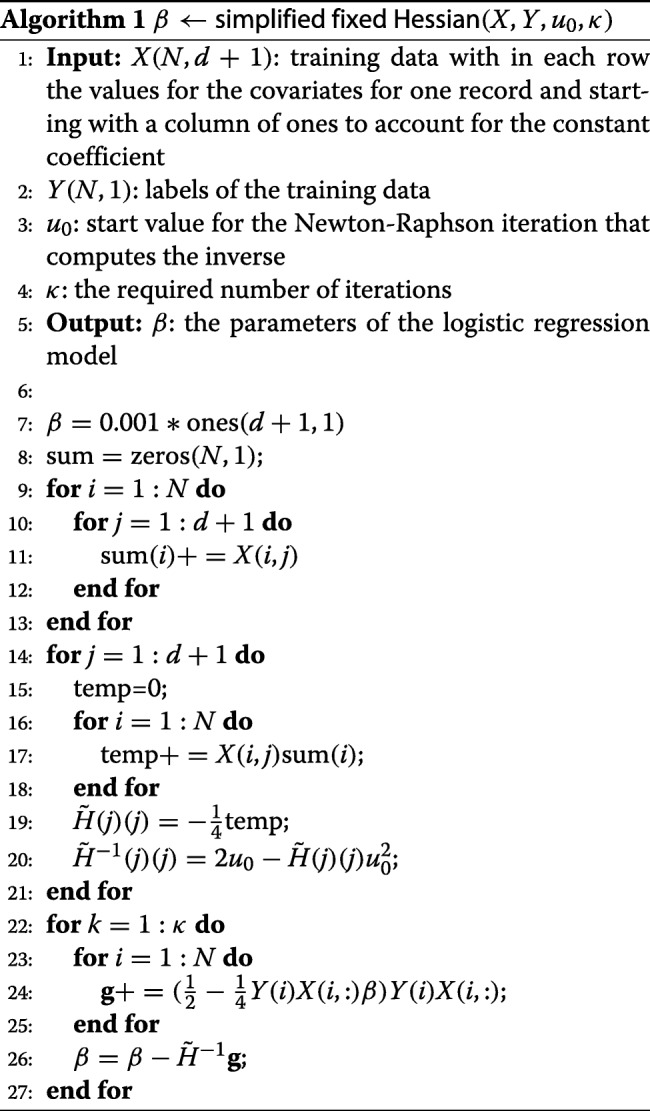


The combination of the above techniques finally results in our simplified fixed Hessian (SFH) method given in Algorithm 1.

We implemented the SFH algorithm in Matlab and verified the accuracy for a growing number of iterations. One can see from Algorithm 1 that each iteration requires 5 homomorphic multiplications, so performing one iteration is quite expensive. In addition, Table 1 indicates that improving the accuracy significantly requires multiple iterations. We will therefore restrict our experiments to one single iteration.

**Table 1:** Performance for the financial dataset with 31 covariates and 700 training records and 19,300 testing records

# iterations	AUC SFH
1	0.9418
5	0.9436
10	0.9448
20	0.9466
50	0.9517
100	0.9599

## Results

### Accuracy of the SFH method

Table 2 shows the confusion matrix of a general binary classifier.

**Table 2:** Comparing actual and predicted classes

		Actual class	
		-1	1
Predicted	-1	True negative (TN)	False negative (FN)
Class	1	False positive (FP)	True positive (TP)

From the confusion matrix, we can compute the true positive rate (TPR) and the false positive rate (FPR) which are given by

6 $$ \text{TPR}=\frac{\#\text{TP}}{\#\text{TP}+\#\text{FN}} \qquad \text{and} \quad \text{FPR}=\frac{\#\text{FP}}{\#\text{FP}+\#\text{TN}} \,.  $$

By computing the TPR and FPR for varying thresholds 0≤*τ*≤1, we can construct the receiver operating characteristic curve or ROC-curve. The ROC-curve is constructed by plotting the (FPR,TPR) pairs for each possible value of the threshold *τ*. In the ideal situation there would exists a point with (FPR,TPR)=(0,1), which would imply that there exists a threshold for which the model classifies all test data correctly.

The area under the ROC-curve or AUC-value will be used as the main indicator of how well the classifier works. Since our SFH method combines several approximations, we need to verify the accuracy of our model first on unencrypted data and later on encrypted data. For well chosen system parameters, there will be no difference between accuracy for unencrypted vs. encrypted data since all computations on encrypted data are exact.

The first step is performed by comparing our SFH method with the standard logistic regression functionality of Matlab. This is done by applying our method with all its approximations to the plaintext data and comparing the result to the result of the “glmfit” function in Matlab. The function *b*=glmfit(*X*,*y*,distr) returns a vector *b* of coefficient estimates for a generalized linear model of the responses *y* on the predictors in *X*, using distribution distr. Generalized linear models unify various statistical models, such as linear regression, logistic regression and Poisson regression, by allowing the linear model to be related to the response variable via a link function. We use the “binomial” distribution, which corresponds to the “logit” link function and *y* a binary vector indicating success or failure to compute the parameters of the logistic regression model with “glmfit”.

**Fig. 1:**
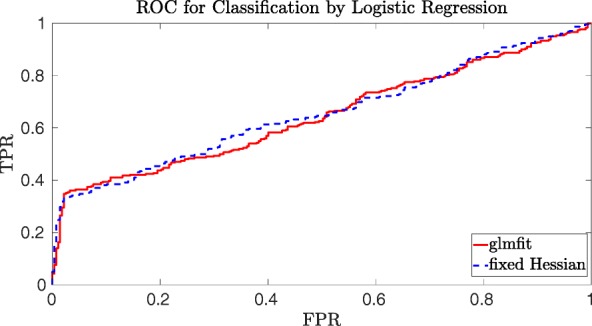
ROC curve for the cancer detection scenario of iDASH with 1000 training records and 581 testing records, all with 20 covariates

**Fig. 2:**
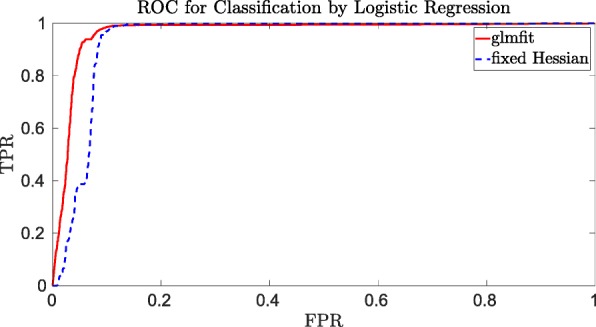
ROC curve for the financial fraud detection with 1000 training records and 19,000 testing records, all with 31 covariates

From Figs. 1 and 2 one can see that the SFH method classifies the data approximately as well as “glmfit” in Matlab, in the sense that one can always select a threshold that gives approximately the same true positive rate and false positive rate. One can thus conclude that the SFH method, with all its approximations, performs well compared to the standard Matlab method, which uses much more precise computations. By computing the TPR and FPR for several thresholds, we found that the approximations of our SFH method shifts the model a bit such that we need a slightly larger threshold to get approximately the same TPR and FPR as for the Matlab model. Since the ideal situation would be to end up with a true positive rate of 1 and false positive rate of 0, we see from Fig. 1 that for the genomics dataset both models are performing rather poorly. The financial fraud use case is, however, much more amenable to binary classification as shown in Fig. 2. The main conclusion is that our SFH method performs almost as well as standard methods such as those provided by Matlab.

### Implementation details and performance

Our implementation uses the FV-NFLlib software library [12] which implements the FV homomorphic encryption scheme. The system parameters need to be selected taking into account the following three constraints:

1. the security of the somewhat homomorphic FV scheme,
2. the correctness of the somewhat homomorphic FV scheme,
3. the correctness of the *w*-NIBNAF encoding.

The security of a given set of system parameters can be estimated using the work of Albrecht, Player and Scott [13] and the open source learning with error (LWE) hardness estimator implemented by Albrecht [8]. This program estimates the security of the LWE problem based on the following three parameters: the degree *D* of the polynomial ring, the ciphertext modulus *q* and $\alpha = \frac {\sqrt {2\pi }\sigma }{q}$ where *σ* is the standard deviation of the error distribution *χ*_err_. The security estimation is based on the best known attacks for the learning with error problem. Our system parameters are chosen to be *q*=2^186^, *D*=4096 and *σ*=20 (and thus $\alpha =\frac {\sqrt {2\pi }\sigma }{q}$) which results in a security of 78 bits.

As explained in the section on the FV scheme, the error in the ciphertext encrypting the result, should be small enough to enable correct decryption. By estimating the infinity norm of the noise we can select parameters that keep this noise under the correctness bound and in particular, we obtain an upper bound *t*_max_ of the plaintext modulus. Similarly, to ensure correct decoding, the coefficients of the polynomial encoding the result must remain smaller than the size of the plaintext modulus *t*. This condition results in a lower bound on the plaintext modulus *t*_min_.

It turns out that these bounds are incompatible for the chosen parameters, so we have to rely on the Chinese Remainder Theorem to decompose the plaintext space into smaller parts that can be handled correctly. The plaintext modulus *t* is chosen as a product of small prime numbers *t*_1_, *t*_2_, …, *t*_*n*_ with ∀*i*∈{1, …, *n*}:*t*_*i*_≤*t*_max_ and $t=\prod _{i=1}^{n}~t_{i} \geq t_{\min }$, where *t*_max_ is determined by the correctness of the FV scheme and *t*_min_ by the correctness of the *w*-NIBNAF decoding. The CRT then gives the following ring isomorphism:

$$R_{t} \rightarrow R_{t_{1}} \times \ldots \times R_{t_{n}}: g(X)\mapsto (g(X)\text{mod}t_{1},~\ldots,~g(X)\text{mod }t_{n})\,. $$

and instead of performing the training algorithm directly over *R*_*t*_, we compute with each of the $R_{t_{i}}$’s by reducing the *w*-NIBNAF encodings modulo *t*_*i*_. The resulting choices for the plaintext spaces are given in Table 3.

**Table 3:** The parameters defining plaintext encoding

		w	t
Genomic data	(1)	71	5179·5189·5197
Financial data	(2)	150	2237·2239

Since we are using the Chinese Remainder Theorem, each record will be encrypted using two (for the financial fraud case) or three (for the genomics case) ciphertexts. As such, a time-memory trade off is possible depending on the requirements of the application. One can choose to save computing time by executing the algorithm for the different ciphertexts in parallel; or one can choose to save memory by computing the result for each plaintext space $R_{t_{i}}$ consecutively and overwriting the intermediate values of the computations in the process.

The memory required for each ciphertext is easy to estimate: a ciphertext consists of 2 polynomials of $R_{q} = \mathbb {Z}_{q}[X]\slash (X^{D}+1)$, so its size is given by 2*D* log2*q* which is ≈186kB for the chosen parameter set. Due to the use of the CRT, we require *T* (with *T*=2 or *T*=3) ciphertexts to encrypt each record, so the general formula for the encrypted dataset size is given by:

$$T (d+1) N 2D \log_{2} q \quad \text{ bits}\,, $$

with *T* the number of prime factors used to split the plaintext modulus *t* and *d*+1 (resp. *N*) the number of covariates (resp. records) used in the training set.

The time complexity of our SFH method is also easy to estimate, but one has to be careful to perform the operations in a specific order. If one would naively compute the matrix $\tilde {H}$ by first computing $\bar {H}$ and subsequently summing each row, the complexity would be *O*(*Nd*^2^). However, the formula of the *k*-th diagonal element of $\tilde {H}$ is given by $\frac {-1}{4}\sum _{j=1}^{d+1}\left (\sum _{i=1}^{N}{x_{k,i}x_{j,i}}\right)$, which can be rewritten as $\frac {-1}{4} \sum _{i=1}^{N}{x_{k,i}} \left (\sum _{j = 1}^{d+1} {x_{j,i}} \right)$. This formula shows that it is more efficient to first sum all the rows of *X* and then perform a matrix vector multiplication with complexity *O*(*Nd*).

This complexity is clearly visible in the tables, more specifically in Tables 4 and 5 for the genomic use case, and Tables 6 and 7 for the financial use case. All these tables show a linear growth of the computation time for a growing number of records or covariates as expected by the chosen order of the computations in the implementation.

**Table 4:** Performance for the genomic dataset with a fixed number of covariates equal to 20

# training records	Computation time	AUC SFH	AUC glmfit
500	22 min	0.6348	0.6287
600	26 min	0.6298	0.6362
800	35 min	0.6452	0.6360
1000	44 min	0.6561	0.6446

The number of testing records is for each row equal to the total number of input records (1581) minus the number of training records

**Table 5:** Performance for the genomic dataset with a fixed number of training records equal to 500 and the number of testing records equal to 1081

# covariates	Computation time	AUC SFH	AUC glmfit
5	7 min	0.65	0.6324
10	12 min	0.6545	0.6131
15	17 min	0.6446	0.6241
20	22 min	0.6348	0.6272

**Table 6:** Performance for the financial dataset with a fixed number of covariates equal to 31

# training records	Computation time	AUC SFH	AUC glmfit
700	30 min	0.9416	0.9619
800	36 min	0.9411	0.9616
900	40 min	0.9409	0.9619
1000	45 min	0.9402	0.9668

The number of testing records is for each row equal to the total number of input records (20,000) minus the number of training records

**Table 7:** Performance for the financial dataset with a fixed number of records equal to 500 and the number of testing records equal to 19,500

# covariates	Computation time	AUC SFH	AUC glmfit
5	5 min	0.8131	0.8447
10	8 min	0.9403	0.9409
15	11 min	0.9327	0.9492
20	15 min	0.9401	0.9629

In Tables 4 and 5 we see that often the AUC value of the SFH model is slightly higher than the AUC value of the glmfit model. However, as mentioned before both models perform poorly on this dataset. Since our SFH model contains many approximations we expect it to perform slightly worse than the “glmfit” model. Only slightly worse because Figs. 1 and 2 already showed that the SFH models classifies the data almost as well as the “glmfit” model. This is consistent with the results for the financial dataset shown in Tables 6 and 7, which we consider more relevant than the results of the genomic dataset due to the fact that both models perform better on this dataset.

## Discussion

The experiments of this article show promising results for the simple iterative method we propose as an algorithm to compute the logistic regression model. A first natural question is whether this technique is generalizable to other machine learning problems. In [14], Böhning describes how to adapt the lower bound method to make it applicable to multinomial logistic regression, it is likely this adaption will also apply to our SFH technique and hence our SFH technique can most likely also be applied to construct a multinomial logistic regression model. In the case of neural networks we can refer to [15]; in order to construct the neural network one needs to rank all the possibilities and only keep the best performing neurons for the next layer. Constructing this ranking homomorphically is not straightforward and not considered at all in our algorithm, hence neural networks will require more complicated algorithms.

When we look purely at the performance of the FV homomorphic encryption scheme, we might consider a residue number system (RNS) variant of the FV scheme as described in [16] to further improve the running time of our implementation. One could also consider single instruction multiple data (SIMD) techniques as suggested in [17] or look further into a dynamic rescaling procedure for FV as mentioned in [6]. These techniques will presumably further decrease the running time of our implementation, which would render our solution even more valuable.

## Conclusions

The simple, but effective, iterative method presented in this paper allows one to train a logistic regression model on homomorphically encrypted input data. Our method can be used to outsource the training phase of logistic regression to a cloud service in a privacy preserving manner. We demonstrated the performance of our logistic training algorithm on two real life applications using different numeric data types. In both cases, the accuracy of our method is only slightly worse than standard algorithms to train logistic regression models. Finally, the time complexity of our method grows linearly in the number of covariates and the number of training input data points.

## Abbreviations

AUC: Area under the curve
CRT: Chinese remainder theorem
FN: False negative
FP: False positive
FPR: False positive rate
FV: Fan vercauteren
KeyGen: Key generation
LWE: Learning with error
RNS: Residue number system
ROC: Receiver operating characteristic
SFH: Simplified fixed Hessian
SIMD: Single instruction multiple data
TN: True negative
TP: True positive
TPR: True positive rate
w-NIBNAF: w non integral base non adjacent form
